# Safety, efficacy, and quality of life with cemiplimab treatment among non-small cell lung cancer patients: a systematic review and meta-analysis

**DOI:** 10.1097/MS9.0000000000003329

**Published:** 2025-04-25

**Authors:** Abhay Mane, Rukesh Yadav

**Affiliations:** a Department of Pharmacology, University of Bedfordshire, UK; b Department of Internal Medicine, Maharajgunj Medical Campus, Tribhuvan University Institute of Medicine, Maharajgunj, Kathmandu, Nepal

**Keywords:** cemiplimab, chemotherapy, non-small cell lung cancer

## Abstract

**Background:**

Immune checkpoint inhibitors have shown promise in treating advanced non-small cell lung cancer and have a solid safety profile. Cemiplimab can be used as monotherapy or in combination with chemotherapy for both squamous and non-squamous cancers. We opted for a systematic review and meta-analysis to find out the efficacy outcome and safety profile along with quality-of-life data of cemiplimab for advanced non-small-cell lung cancer (NSCLC).

**Methods:**

A rigorous search of literature on PubMed, Embase, and Google Scholar was done to find relevant published publications till December 1, 2023. Outcomes like objective response rate (ORR), overall survival (OS), progression-free survival (PFS), common side effects, and quality of life among the cemiplimab and the control group were used to conduct meta-analysis using the fixed/random effect model for combined odds ratio (OR), combined hazard ratio (HR) and mean difference at confidence interval (CI) of 95%.

**Results:**

Two randomized clinical trials EMPOWER 1 and EMPOWER 3 with 1092 advanced NSCLC patients along with their follow-up studies were included. There was significantly higher OS (HR = 0.62 [0.54, 0.70], *P* < 0.0001) and PFS (HR = 0.54 [0.48, 0.60], *P* < 0.0001] in the treatment group. Similarly, the combined ORR was significantly higher in the treatment group as compared to the control group (2.63 [95% CI: 2.17–3.20, *P* ≤ 0.001]). Treatment-emergent adverse effects were not different between the groups. Finally, the quality-of-life scores between the two groups were nonsignificant.

**Conclusion:**

With regard to OS, PFS, ORR, quality of life scores, and an acceptable safety profile in advanced non-small cell lung cancer, cemiplimab showed clinically relevant and statistically significant improvements making it standard of care for such patients.

## Introduction

Lung cancer is the most prevalent cancer-related cause of death worldwide, taking the lives of almost 1.6 million people annually It is estimated that non-small cell lung cancer (NSCLC) accounts for 85% of these deaths^[^1^]^. Mutations in the epidermal growth factor receptor (EGFR) genes are the most common cause of NSCLC, which accounts for roughly 75%–80% of all lung malignancies^[^2^]^. When NSCLC is diagnosed as stage 3 or stage 4, it is considered advanced (NSCLC stages III and IV). There are two forms: locally advanced NSCLC and metastatic NSCLC. Locally advanced NSCLC, when NSCLC is diagnosed as stage 3, occurs when the cancer may have spread to the regional lymph nodes. Metastatic NSCLC, when NSCLC is diagnosed as stage 4, occurs when the cancer has spread to other organs such as the other lung, brain, liver, or to other parts of the body^[^3^]^.

**HIGHLIGHTS**

- Immune checkpoint inhibitors have shown promise in treating advanced non-small cell lung cancer and have a solid safety profile.
- We opted for a systematic review and meta-analysis to find out the efficacy outcome and safety profile along with quality-of-life data of cemiplimab for non-small cell lung cancer.
- There was significantly higher overall survival, progression-free survival and objective response rate in the treatment group as compared to the healthy control group.
- No significant differences in the treatment-emergent adverse effects and quality of life were observed among the treatment and the control groups.
- For advanced non-small cell lung cancer, cemiplimab monotherapy as a first line or in combination can therefore be regarded as the standard of care.

While platinum-based chemotherapy has been the conventional approach for the treatment of advanced NSCLC for a considerable amount of time, it has also been linked to a poor response rate, as well as short overall and progression-free survival (PFS)^[^4^]^.

Immune checkpoint inhibitors (ICIs) have shown promise in treating advanced NSCLC and have a solid safety profile. Among the ICI targets are programmed cell death protein-1 (PD-1), programmed death ligand 1 (PD-L1), and cytotoxic T-lymphocyte-associated antigen 4^[^5^]^. When it comes to treating metastatic NSCLC tumors that lack gene fusions in the ALK and ROS genes or driving mutations in the EGFR gene, immunotherapy, either as monotherapy or in combination with chemotherapy, has solidified its place as the first line of treatment.

The efficacy of cemiplimab was demonstrated in the EMPOWER-Lung 1 and EMPOWER-Lung 3 trials^[^6,7^]^, respectively. Cemiplimab-rwlc is a highly potent hinged, stabilized human IgG4 monoclonal antibody that targets PD-1 and FDA-approved immunotherapy option for treating metastatic NSCLC. It can be used as monotherapy or in combination with chemotherapy for both squamous and non-squamous histologies^[^8^]^.

Tumors negative for EGFR, ALK, and ROS1 can have significant levels of PD-L1 expression (tumor proportion score ≥50%) detected by cemiplimab. Cemiplimab monotherapy is a first-line treatment option for metastatic or locally advanced NSCLC patients who are not eligible for surgical resection or definitive chemoradiation^[^9^]^.

In the clinical trials, cemiplimab has demonstrated encouraging outcomes. This study has the main aim to review the drug’s efficacy outcomes along with quality of life (QoL), the follow-up data, and also adverse effects in advanced NSCLC and gather further evidence to support this. Therefore, our research question focuses to compare the treatment group and control group through various outcomes.

## Methodology

The Preferred Reporting Items for Systematic Reviews and Meta-Analyses (PRISMA) declaration 2020 is followed in the reporting of this systematic review and meta-analysis^[^10^]^. The review has been registered in Prospero register for systematic reviews.

The research question for this review was to assess clinical safety and effectiveness of cemiplimab alone or with combined chemotherapy for treating advanced or previously treated NSCLC based on outcomes like objective response rate (ORR), overall survival (OS), PFS, common side effects and QoL. We then wrote the criteria for inclusion and exclusion. The percentage of patients who experienced both a partial and complete response (CR and PR) was used to establish the ORR. The fraction of patients who had stable disease (SD), PR, and CR was called the disease control rate. SD is defined as neither enough rise to be classified as progressive disease (PD) nor enough shrinkage to be classified as PR. On the other hand, PD is defined as a minimum 20% increase in the total of the target lesions’ longest diameters. ORR is defined as the percentage of patients who, based on response evaluation criteria for solid tumors, had either a full or partial response to treatment.

PFS was defined as the time interval between the beginning of treatment and the earliest possible event; death or the start of the disease’s deterioration. OS was defined as the amount of time that passed between the patient’s death from any cause and the beginning of cemiplimab medication^[^11^]^.

### Study inclusion and exclusion criteria

The following requirements should have been met by the inclusion criteria for the studies that were chosen: (1) Studies that used PD-1 inhibitor cemiplimab monotherapy or combination to treat advanced NSCLC 2) data on one of the outcomes like ORR, OS, PFS, adverse effects, and QoL. (3) Observational studies and randomized control trials. (4) Studies only in English.

The following were the exclusion criteria: (1) Research on animals or *in vitro* (2) inadequate information (3) redundant content (4) reviews or meta-analytic studies (5) studies not in English.

### Search methods and study selection

The English language literature was searched in the PubMed, Embase, and Google Scholar databases from the time of their creation until December 1, 2023. A database search was performed using Boolean logic, and the search phrases “PD-1 inhibitor,” “Cemiplimab,” “Non-Small Cell Lung Cancer,” and “NSCLC” were linked together using the Boolean search operators “AND” and “OR.” We also looked through each included study’s reference list to find more potentially relevant articles. The initial search yielded articles. After that, duplicates were suitably eliminated from all of the studies that made the short list and imported into ENDNOTE V.6. The papers were first separately examined by the two authors based just on the title, keywords, and abstract. After a discussion, the studies were further confirmed. Following the first screening, two reviewers examined the articles in detail. Through discussion, we were able to address the disagreements over the final research selection between the two primary reviewers. Finally, seven articles in total were included in the review. The population’s possible overlap was assessed generally based on the authorship, hospital environment, and recruitment time. Higher quality research or bigger sample sizes were included when there was overlap.

### Data extraction

After carefully examining the selected publications that fit our inclusion criteria, two independent authors extracted precise data on various topics under two tables for the systematic review that included baseline features and clinical outcomes were recorded in Microsoft Excel 2013 (Microsoft Corp, Redmond, USA). Under baseline features, the headings were Author/Published Year, Study site, Study design, Study period, Participants, Mean age (in years), Sex (female/male), and Duration of treatment (Table 1). Under clinical outcomes, the headings were Author/Published year, Cemiplimab either monotherapy or combination with its formulation, and outcomes after like ORR, OS, PFS, adverse effects, and QoL.

**Table 1:** Features of studies included in the review

Study name	Trial included	Number of patients	Intervention	Outcomes	Adverse outcomes
Sezer *et al*^[^6^]^	EMPOWER 1 trial	Cemiplimab-283, chemotherapy-280	Cemiplimab 350 mg every 3 weeks or platinum-doublet chemotherapy	OS, PFS, ORR, CR, PR, SD, and NPD, Kaplan–Meir curve	Grade ≥3: 50/355 vs 134/342
					Mortality: 34/355 vs 31/342
Moreno *et al*^[^16^]^	NCT02383212	20 patients with cemiplimab	Cemiplimab 200 mg 2 weekly intravenously for upto 48 weeks	CR, PR, SD, PD, ORR	Grade ≥3 TEAEs: 12/20
Gogishvili *et al*^[^7^]^	EMPOWER 3 trial	Cemiplimab-312 or placebo-154	Cemiplimab 350 mg or placebo	OS, ORR, CR, PR, SD, PD, Kaplan–Meir curve	Grade ≥3
					TEAEs: 136/312 vs 48/153
			3 weekly for up to 108 weeks in combination with 4 cycles of platinum-doublet chemotherapy pemetrexed maintenance as indicated		Mortality: 19/312 vs 12/153
Gümüş *et al*^[^13^]^	EMPOWER 1	Cemiplimab-283; chemotherapy-280	Cancer Quality of Life-Core 30 (QLQ-C30) and Lung Cancer Module (QLQ-LC13) questionnaires	QLQ-C30, QLQ-LC13 scores	Reduced likelihood of treatment-related (dyspnea, cough, chest pain, pain in other body parts, fatigue) and disease-related (dyspnea) symptoms in cemiplimab
Makharadze *et al*^[^14^]^	EMPOWER 3 trial	Cemiplimab 350 mg-312; or placebo-154	Cancer Quality of Life-Core 30 (QLQ-C30) and Lung Cancer Module (QLQ-LC13) questionnaires	GHS/QoL, delays in TTD	Statistically significant TTD delays, all in favor of chemotherapy plus cemiplimab
Makharadaze *et al*^[^12^]^	EMPOWER 3 part 2: 2-year follow-up	Cemiplimab 350 Mg-312) or placebo-154	Cemiplimab 350 mg or placebo	ORR, OS, PFS, CR, PR, SD, ORR, Kaplan–Meir curve	Grade ≥3
					TEAEs: 152/312 vs 50/153
					Mortality: 27/312 vs 14/153
			3 weekly for up to 108 weeks in combination with 4 cycles of platinum-doublet chemotherapy (followed by pemetrexed maintenance as indicated at 2-year follow-up		
Özgüroğlu *et al*^[^15^]^	EMPOWER 1 trial: 35-month follow-up	Cemiplimab-284, chemotherapy-281	Intravenous cemiplimab 350 mg	OS, PFS, ORR, CR, PR, SD, and NPD, Kaplan–Meir curve	Grade ≥3
					TEAEs: 65/361 vs 137/343
			3 weekly for up to 108 weeks, or until disease progression		
					Mortality: 10/356 vs 7/343
			Versus chemotherapy		

CR, complete response; GHS, global health status; NPD, nonprogressive disease; ORR, objective response rate; ORR, objective response rate; OS, overall survival; PFS, progression-free survival; PR, partial response; QoL, quality of life; SD, stable disease; TEAE, treatment-emergent adverse events.

Email correspondence was used to get in touch with the associated authors of the relevant studies if any necessary data were missing, not published in the study, or reported in an unusual format. In such circumstances, additional supplementary material related to the main publication was also investigated.

The bias risk evaluation tool from the Cochrane Collaboration was utilized as a standardized critical appraisal instrument to evaluate the bias risk among the included studies (https://training.cochrane.org/handbook/current). The bias risk was separately examined by two reviewers based on incomplete outcome data, selective result reporting, blinding of participant personnel and outcome assessors, allocation concealment, sequence generation, and other potential sources of bias. Discussions were used to settle disagreements.

### Data synthesis and analysis

The effect measures were combined odds ratio (OR) and combined hazard ratio (HR) for the outcomes OS, PFS, ORR, CR, PR, SD, PD, mortality, and grade ≥3 treatment-emergent adverse effects (TEAEs) among the treatment and the control groups. Furthermore, for the quality of measures, mean differences in QoL scores among the treatment and the control group were calculated. The analysis was performed using the software Review Manager 5.4. Forest plots were generated to depict the results of the final analysis. The Higgins *I*^2^ statistic was used to evaluate the statistical heterogeneity with values over 50% suggesting moderate heterogeneity, and over 75% suggesting severe heterogeneity. Statistical significance was considered when the *P*-value was below 0.05. The random effect model was applied whenever *I*^2^ was above 50%, otherwise fixed effect model was applied for the meta-analysis.

## Results

### Search results and study selection

**Figure 1.**
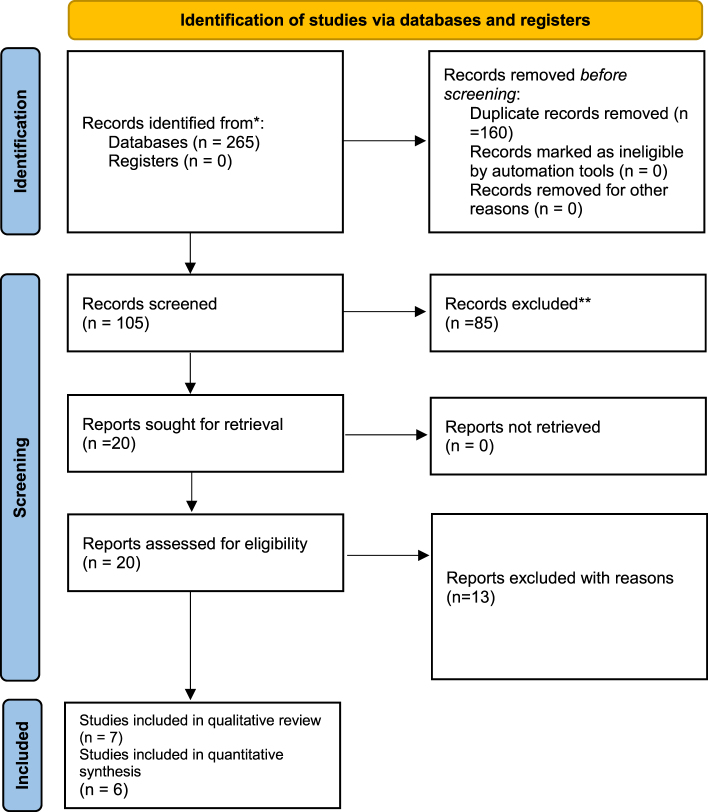
PRISMA diagram showing the selection process and final included studies.

Through electronic database searches, we found 265 papers; manual searches of reference lists and relevant systematic reviews yielded no more research. We used the titles and abstracts to filter 105 articles after removing duplicates. Following screening, 20 full-text publications were located and evaluated in accordance with the predetermined inclusion criteria, resulting in the eligibility of 7 papers for the review^[^6,7,12–16^]^. Figure 1 displays the PRISMA diagram that explains the identification and selection procedure.

### Baseline demography

Two randomized clinical trials EMPOWER 1 and EMPOWER 3 with 1029 advanced NSCLC patients were included^[^6,7^]^. EMPOWER 1^[^6^]^ was a, global, open-label phase 3 randomized clinical trial. Whereas, EMPOWER 3^[^7^]^ was multi-center, controlled, double-blind phase 3 randomized clinical trial. Remaining studies were the follow-up studies of these two trials. EMPOWER 1^[^6^]^ compared cemiplimab with chemotherapy^[^6^]^ while EMPOWER 3^[^7^]^ compared cemiplimab and chemotherapy with placebo and chemotherapy. The median age of the participants in both groups was similar in the 50s–70s. Cemiplimab 350 mg intravenously and three weekly in both trials while one expansion cohort used cemiplimab 200 mg intravenously every 2 weeks. Male predominance and Asian minority were in both trials. Non-squamous type of NSCLC was predominant in both trials. EMPOWER 1 study included either smokers or past-smoker with advanced NSCLC while other trials included both smokers and non-smokers. A total of 99 patients had brain metastasis, 873 had metastatic stage and 156 were locally advanced cases. The Eastern Cooperative Oncology Group performance status score was 0 in 221 patients and 1 in 804 patients in both groups. Additionally, 82 NSCLC patients in both trials had received previous cancer treatment.

In an exploratory analysis of the EMPOWER Lung 1 trial, Özgüroğlu *et al*^[^15^]^ reported results at 35 months of follow-up as well as the impact of combining chemotherapy with cemiplimab at the time of disease progression. Similarly, Makharadze *et al*^[^14^]^ reported protocol-specific final OS analysis and other outcomes of 2 years follow-up with analysis of subgroup by tumor histology and level of PD-L1 expression. Conversely, Moreno *et al*^[^16^]^ published the results of an expanded cohort consisting of 20 cemiplimab-treated patients.

Both trials had extension studies to report patient-related outcomes through the European Organization for Research and Treatment of Cancer Quality of Life-Core 30 and Lung Cancer Module (QLQ-LC13) questionnaires and also time to definitive clinically meaningful deterioration (TTD) analysis performed for global health status (GHS) and QoL^[^12,13^]^.

### Risk of bias

**Figure 2.**
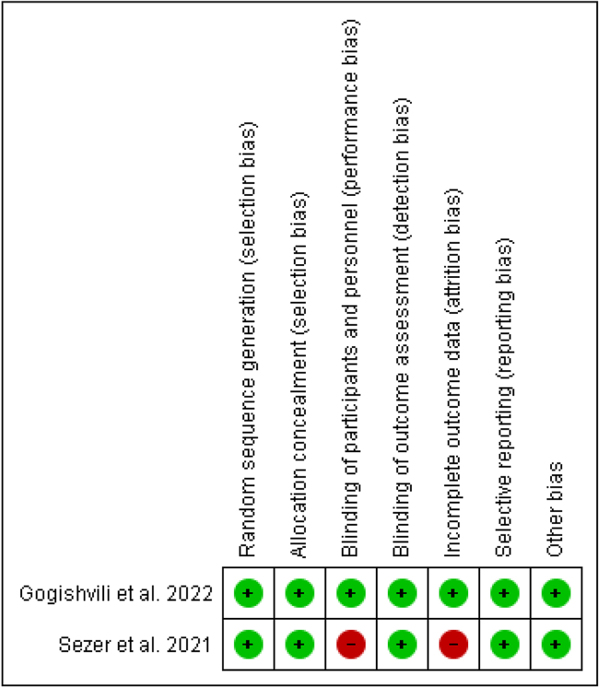
Risk of bias summary: review authors’ judgments about each risk of bias item for each included trial (EMPOWER 1 and EMPOWER 3).

The EMPOWER 1 trial has a high bias risk in blinding participants and personnel and in incomplete outcome data while low bias risk in every other domain. While the EMPOWER 3 trial has a low bias risk in every domain (Fig. 2).

### Survival analysis outcomes

#### Overall survival

**Figure 3.**
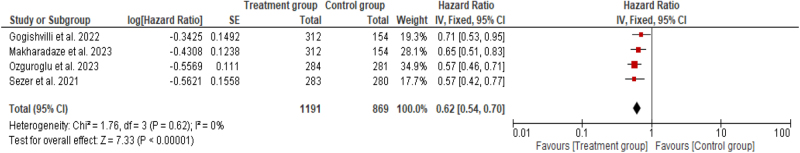
Forest plot showing pooled hazard ratio with 95% CI for OS.

Four studies^[^6,7,14,15^]^ totaling 1191 patients in the treatment group and 869 patients in the control group were included in our analysis, and the results indicated that the treatment group had a considerably higher OS than the control group (*P* < 0.0001, HR = 0.62 [0.54, 0.70]) and no heterogeneity was found in the analysis (*P* = 0.62, *I*^2^ = 0%) (Fig. 3).

#### Progression-free survival

**Figure 4.**
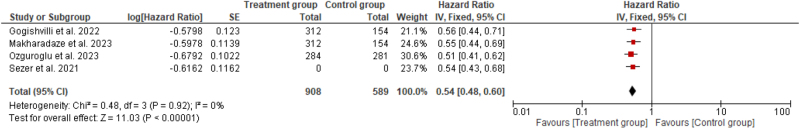
Forest plot showing pooled hazard ratio with 95% CI for PFS.

Our analysis revealed that the treatment group had significantly greater PFS than the control group, based on a total of four studies^[^6,7,14,15^]^ with 908 patients in the treatment group and 589 patients in the control group (*P* < 0.0001, HR = 0.54 [0.48, 0.60]). No heterogeneity was found in the analysis (*P* = 0.92, *I*^2^ = 0%) (Fig. 4).

#### Clinical efficacy

The treatment responses in these studies were found to be:

#### Objective response rate

**Figure 5.**
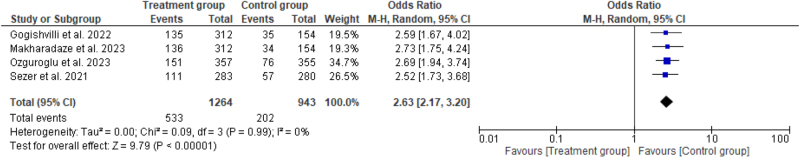
Forest plot showing pooled odds ratio with 95% CI for ORR.

Four studies^[^6,7,14,15^]^ compared ORR between treatment groups (*n* = 533) and control group (*n* = 202). The pooled OR for ORR was 2.63 (95% CI: 2.17–3.20, *P* ≤ 0.001) with significant findings. No significant heterogeneity was revealed in the analysis (*I*^2^ = 0%, *P* = 0.99) (Fig. 5).

#### Complete response

**Figure 6.**
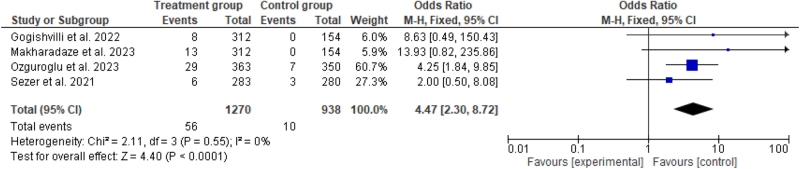
Forest plot showing pooled odds ratio with 95% CI for CR.

Four studies^[^6,7,14,15^]^ compared CR between treatment groups (*n* =56 and the control group (*n* = 10). The pooled OR for CR was 4.47 (95% CI: 2.30–8.72, *P* ≤ 0.001) with significant findings. No significant heterogeneity was revealed in the analysis (*P* = 0.55, *I*^2^ = 0%) (Fig. 6).

#### Partial response

**Figure 7.**
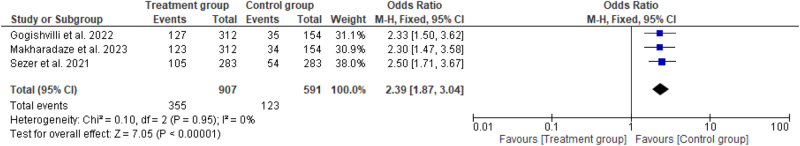
Forest plot showing pooled odds ratio with 95% CI for PR.

Three studies^[^6,7,14^]^ compared PR between treatment groups (*n* = 355) and the control group (*n* = 123). The pooled OR for PR was 2.39 (95% CI: 1.87–3.04, *P* ≤ 0.001) with significant findings. No significant heterogeneity in the analysis was revealed (*I*^2^ = 0%, *P* = 0.95) (Fig. 7).

#### Stable disease

**Figure 8.**
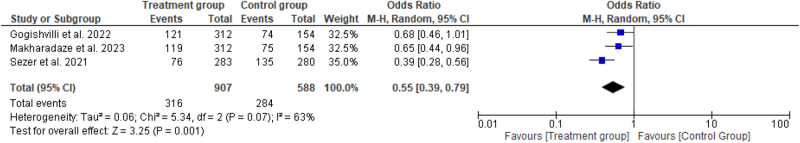
Forest plot showing pooled odds ratio with 95% CI for SD.

Three studies^[^6,7,14^]^ compared SD between treatment groups (*n* = 316) and the control group (*n* = 284). The pooled OR for SD was 0.55 (95% CI: 0.39–0.79, *P* = 0.001) with significant findings. Significant heterogeneity was noted in the analysis that validated the use of random effect model in the analysis (*I*^2^ = 63%, *P* = 0.07) (Fig. 8).

#### Progressive disease

**Figure 9.**
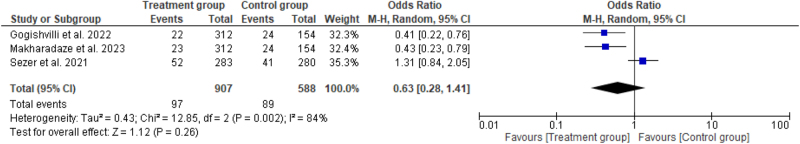
Forest plot showing pooled odds ratio with 95% CI for PD.

Three studies^[^6,7,14^]^ compared PD between treatment groups (*n* = 97) and the control group (*n* = 89). The pooled OR for PD was 0.63 (95% CI: 0.28–1.41, *P* = 0.26) with no significant findings. Significant heterogeneity was noted in the analysis that validated the use of random effect model in the analysis (*I*^2^ = 84%, *P* = 0.002) (Fig. 9).

### Safety evaluation

#### Grade ≥ treatment-emergent adverse effects

**Figure 10.**
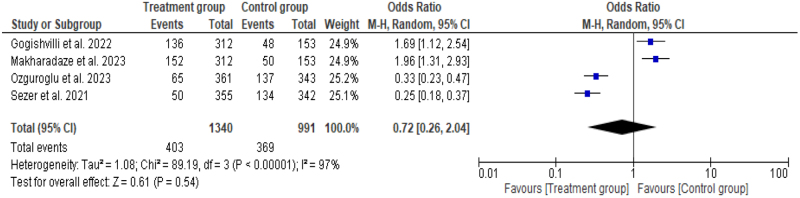
Forest plot showing pooled odds ratio with 95% CI for grade ≥3 TEAEs.

Four studies^[^6,7,14,15^]^ compared adverse effects of grade ≥3 between treatment groups (*n* = 403) and the control group (*n* = 369). The pooled OR for adverse effects was 0.72 (95% CI: 0.26–2.04, *P* = 0.54) with no significant findings. Significant heterogeneity was noted in the analysis that validated the use of random effect model in the analysis (*I*^2^ = 97%, *P* ≤ 0.0001) (Fig. 10).

#### Mortality

**Figure 11.**
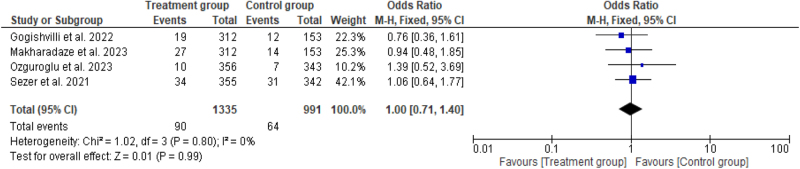
Forest plot showing pooled odds ratio with 95% CI for mortality.

Four studies^[^6,7,14,15^]^ compared mortality rates between treatment groups (*n* =90 and the control group (*n* = 64). The pooled OR for mortality was 1.00 (95% CI: 0.71–1.40, *P* = 0.99) with no significant findings. There was no significant heterogeneity in the analysis so that fixed effect model was reasonable for the analysis (*I*^2^ = 0%, *P* = 0.80) (Fig. 11).

### Quality of life

#### Quality of Life Questionnaire-C30 scores

**Figure 12.**
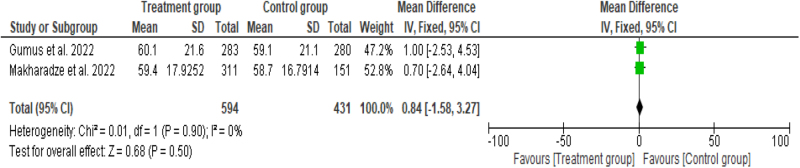
Forest plot showing the mean difference with 95% CI for QLQ-C30 scores.

Two studies^[^12,13^]^ compared the mean Quality of Life Questionnaire-C30 (QLQ-C30) scores between the groups. The mean difference between the treatment groups (*n* = 594) and control group (*n* = 431) was found to be 0.84 (95% CI: −1.58 to 3.27, *P* = 0.50) with no significant finding. No significant heterogeneity was revealed in the analysis (*I*^2^ = 0%, *P* = 0.90) (Fig. 12).

## Discussion

Our systematic review and meta-analysis is the first one to discuss the safety and efficacy of cemiplimab for the management of advanced NSCLC. In the studies included in review, the treatment group comprised of cemiplimab with/without the chemotherapy and the control group comprised placebo with/without chemotherapy. The cemiplimab group had good clinical outcomes, reasonable survival outcomes, good QoL scores, and acceptable safety profiles in comparison to the control group.

In metastatic NSCLC, PD-1, and PD-L1 inhibitors are now the mainstay of initial treatment for tumors without EGFR driving mutations or ALK and ROS gene fusions. These inhibitors can be used alone or in combination with chemotherapy^[^8^]^. Cemiplimab-rwlc is a monoclonal antibody (IgG4) that is highly potent, hinged, stabilized, and directed against PD-1. Based on the results of the EMPOWER-Lung 1 and EMPOWER-Lung 3 trials, for the treatment of metastatic NSCLC, cemiplimab is a recently authorized immunotherapy regimen in 2022. Cemiplimab alone and in combination with chemotherapy was authorized by US FDA in 2021 and 2022, respectively. It can be used as single therapy or in combination regimen with chemotherapy^[^6,7^]^. Cemiplimab’s prescription in the US excludes the patients with both EGFR+/ALK + NSCLC and ROS1 + NSCLC due to the two EMPOWER lung trials that did not include both types of NSCLC^[^17^]^. Sezer *et al*^[^6^]^ only recruited smokers or ex-smokers, 11% of patients were from Asian sites, and the majority of patients were male (88% in the cemiplimab group and 83% in the chemotherapy group). These are the main differences between the two trials^[^6^]^.

In the EMPOWER 1 trial, for cemiplimab, there was no attainment of the median OS, while for chemotherapy, median OS was 14.2 months (HR = 0.57, CI = 0.42–0.77; *P* = 0.002).

The PFS (median) for cemiplimab was 8.2 versus 5.7 months for the chemotherapy (HR = 0.54, CI = 0.43–0.68; *P* ≤ 0.0001). All subgroups studied, including age groups (60 years and over), squamous and non-squamous histology, locally progressed and metastatic cancer, and the presence of brain metastases, showed benefits of cemiplimab for OS and PFS, with the exception of OS in female patients^[^6^]^. Similar to this, in a 3-year follow-up, the updated median PFS was 8.1 months in the cemiplimab versus 5.3 months in the chemotherapy group (HR = 0.51; CI = 0.42–0.62; *P* < 0.0001) and the updated median OS was 26.1 months in the cemiplimab arm versus 13.3 months in the chemotherapy arm (HR = 0.57; CI = 0.46–0.71; *P* < 0.0001)^[^15^]^.

In a similar way, the PFS in the EMPOWER 3 trial was 8.2 months for the group receiving cemiplimab plus chemotherapy and 5.0 months for the group receiving placebo plus chemotherapy (HR = 0.56; CI = 0.44–0.70; *P* < 0.0001). The median OS for the chemotherapy plus placebo group was 13.0 months, whereas the OS for the cemiplimab + chemotherapy group was 21.9 months (HR = 0.71; CI = 0.53–0.93; *P* = 0.014)^[^7^]^. A 2-year follow-up showed a median progression-free survival of 8.2 versus 5.5 months (HR = 0.55; CI = 0.44–0.68; *P* < 0.0001) and a median overall survival of 21.1 months versus 12.9 months (HR = 0.65 [0.51–0.82]; *P* = 0.0003)^[^14^]^. Our analysis also showed cemiplimab lead to significantly higher OS and PFS. However, chemotherapy addition might cause slightly different results.

In the EMPOWER 1 trial, the cemiplimab group showed an objective response of 39% compared to 20% for the chemotherapy group. The OR was 2.53 (95% CI 1.74–3.69; *P* < 0.0001)^[^6^]^. In further follow-up, showed ORR of 46.5% versus 21.0% (OR 3.26; *P* < 0.0001)^[^15^]^. In EMPOWER 3 trial, Cemiplimab plus chemotherapy showed ORR of 43.3% compared to 22.7% for the placebo plus chemotherapy (OR = 2.68 [1.72–4.19]; *P* < 0.0001)^[^7^]^. Similarly, in the 2-year follow-up, ORR was 43.6% versus 22.1%^[^14^]^. A phase 1 expansion cohort among 20 patients showed that five had PR, four had SD and 10 had PD while the ORR was 25.0%^[^16^]^. In our analysis, ORR was found significantly high in the cemiplimab group than in the control group. Similarly, outcomes like CR, PR, and SD showed significant data in the treatment group while for PD no significant findings were seen. In a subgroup analysis of locally advanced NSCLC from two trials, 15% of patients in each trial received treatment for locally advanced NSCLC. During the follow-up at 3 years, cemiplimab monotherapy produced a median overall survival of 26.1 versus 13.9 months, PFS of 8.1 versus 6.2 months, and ORR of 49% versus 31% in EMPOWER-Lung 1.

At a follow-up of 2 years, the median OS in EMPOWER-Lung 3 was 24.1 months, compared to 13.8 months. The median progression-free survival was 12.5 months, compared to 6.2 months, and the ORR was 58% versus 29% showing benefits in patient with unresectable NSCLC patients not eligible for concurrent chemoradiotherapy^[^18^]^. Similarly, cemiplimab monotherapy had better effectiveness outcomes than chemotherapy in patients with brain metastases, including longer median overall survival, longer median progression-free survival, a higher objective response, and a longer median duration of efficacy^[^19^]^. This shows a wide variety of benefits of cemiplimab.

Sustaining QoL is crucial for terminal NSCLC, particularly when assessing the effectiveness of novel combined therapy regimens that may have additional side effects. The results of two studies that examined patient-related outcomes revealed that when compared to the chemotherapy group, the cemiplimab group showed a significant reduction in overall pain symptoms, delayed the time to definitive clinically meaningful deterioration (TTD) in lung carcinoma-specific and tumor-related symptoms and functions, and an improvement in survival that was associated with improvements in functioning and GHS or QoL score^[^12,13^]^. Our analysis found no significant mean difference in QOL-Q C-30 score between the two groups. The paucity of studies in the analysis might be responsible for this nonsignificant reason.

The most frequent grades III–IV treatment-related adverse events in the EMPOWER 1 trial were anemia and neutropenia in patients who received chemotherapy, and elevated aspartate aminotransferase and pneumonia in individuals receiving cemiplimab^[^6^]^. Anemia and neutropenia were higher in both groups in the EMPOWER 3 trial and in the follow-up data^[^7^]^. Cemiplimab was linked with a more immune-associated side events than chemotherapy. The study of Cemiplimab in advanced NSCLC(EMPOWER trials), the immune-associated side effects profile seems consistent, and it might even be higher than those previously documented for cemiplimab in other tumor types^[^20,21^]^. In a meta-analysis, overall, 1.24% of PD-L1 inhibitor-associated fatal side effects occurred; this was significantly greater in non-squamous NSCLC, phase I trial, and among the group of middle-aged people while comparing to the equivalent reference group. Among the causes of death, pulmonary devastating side effects were the most common^[^22^]^. In the^[^6^]^ EMPOWER 1 trial, autoimmune myocarditis, heart failure, cardiopulmonary failure, cardiac arrest, nephritis, respiratory failure, and sepsis with shock were the causes of mortality in the cemiplimab group. In the follow-up studies also no new safety signals were identified^[^14,15^]^. Our analysis found a nonsignificant association of adverse events and mortality between treatment groups and control groups. This defines cemiplimab as a safer option in advanced NSCLC.

### Strength and limitations

This systematic review and meta-analysis is the first of its kind to analyze the safety and efficacy profiles of cemiplimab along with follow-up findings and QoL parameters when compared with the control group. Furthermore, the inclusion of the randomized clinical trials as the only study type ensures the quality of this review. Three of the included studies were randomized, multi-center, controlled, double-blind, and phase three clinical trials. The remaining four were randomized, multi-center, open-label phase three clinical trials. There are various limitations in this research. First, only data from two major trials and their follow-up data were eligible for inclusion in this study. Second, was the variability of participants in the two included trials. Third was the inability to present publication bias due to fewer studies. Fourth was high heterogeneity in some of the analyses. The final one was the use of cemiplimab alone or in combination with chemotherapy. For example, four studies compared cemiplimab with chemotherapy plus placebo while the three studies compared cemiplimab plus chemotherapy with placebo plus chemotherapy. Even though the chemotherapy regimen used was similar in all the included studies, the mentioned combination pattern might have influenced the outcomes and hence the analysis results.

## Conclusion

Cemiplimab, either as a first-line monotherapy or in combination, can be regarded as the standard of care for patients with advanced NSCLC who have 50% PD-L1 expression but no targetable mutations. In comparison to the control group, cemiplimab therapy showed a clinically relevant and statistically significant improvement in OS, PFS, and ORR only but not QOL scores. Moreover, evidence suggests that the safety profile of cemiplimab was equivalent to platinum-based chemotherapy. In future prospective research and clinical trials, more validations focusing on a varied population across different histological types and varying degrees of PD-L1 expression are required.

## Data Availability

Not applicable.

## Research registration unique identifying number (UIN)

PROSPERO Registration ID: CRD42024559222.

## Author agreement statement

The manuscript has been read and approved by all the authors and the requirements for authorship have been met, and each author believes that the manuscript represents honest work.
